# Trajectories of Bystander Behaviors in Bullying during Secondary Education: the Role of Moral Disengagement and Conformity To Peer Pressure

**DOI:** 10.1007/s10964-025-02276-8

**Published:** 2025-10-22

**Authors:** Paula García-Carrera, Rosario Ortega-Ruiz, Antonio Camacho, Claire F. Garandeau, Christina Salmivalli, Eva M. Romera

**Affiliations:** 1 Department of Psychology, Universidad de Córdoba, Córdoba, Spain; 2 INVEST Research Flagship, Department of Psychology, University of Turku, Turku, Finland

**Keywords:** Adolescence, Defending, Pro-bullying, Passive, Parallel- process LCGA

## Abstract

Bystanders shape bullying dynamics, yet little is known about how their behaviors change over time and related socio-moral factors. This study adopted a person-centered longitudinal approach to examine joint trajectories of bystander behaviors and their co-evolution with moral disengagement and conformity to peer pressure. A sample of 994 Spanish adolescents (48% girls; *M*_age T1_ = 12.5, *SD* = 0.6) was surveyed across three waves. Parallel-process Latent Class Growth Analysis identified three trajectories: *Sustained Defending* (defending declined slightly at the end of secondary education), *Increase Pro-bullying* (pro-bullying gradually increased), and *Decrease Pro-bullying* (pro-bullying decreased from the first wave). These behavioral shifts were linked to specific socio-moral mechanisms: in the *Increase Pro-bullying* profile, persistently high moral disengagement and rising conformity co-occurred with the transition from passivity to pro-bullying, while in the *Decrease Pro-bullying* profile, reductions in both processes co-occurred with the shift toward defending. Findings provide evidence of the variability in behaviors adopted by bystanders across adolescence, with a significant change in pro-bullying patterns from 8^th^ to 9^th^ Grade. The identification of bystanders’ trajectories and the coevolution of sociomoral processes support the reconceptualization of bystanding as a dynamic developmental phenomenon shaped by intrapersonal and interpersonal forces and highlighting targets for long-term intervention.

**Supplementary Information:**

The online version contains supplementary material available at 10.1007/s10964-025-02276-8.

## Introduction

Bullying is a deliberate and repeated form of aggression involving a real or perceived power imbalance, typically aimed at undermining a peer’s identity, emotional safety, or sense of belonging within the group (Hellström et al., 2021). Beyond its behavioral manifestations, bullying operates within broader peer group dynamics and is shaped by relational and moral processes that sustain asymmetries of power in social interactions (Ortega-Ruiz, 2020). These processes often result in lasting emotional and psychological consequences not only for victims, but also for those who witness or are indirectly involved in the aggression (Schoeler et al., 2018). During secondary education, typically between the ages of 12 and 16 in the Spanish context, peer group salience and status concerns make adolescents particularly sensitive to social dynamics that either reinforce or challenge bullying (Pouwels et al., 2019). Bystanders play a key role in these dynamics. Rather than being mere observers, they often adopt distinct roles, defending, passive, or pro-bullying (Salmivalli, 2010). While approximately 74% of students take on one or more of these roles (Pouwels et al., 2017), the majority fail to intervene (Hawkins et al., 2001). Traditionally, these roles have been conceptualized as stable and mutually exclusive. However, growing evidence suggests that individuals may exhibit variability in their responses across time, reflecting contextual and relational influences (Huitsing et al., 2014). While some adolescents move between roles, research shows that the majority tend to follow developmentally normative patterns of prosocial behavior, particularly during adolescence, when concern for fairness, empathy, and peer wellbeing typically increases (Pozzoli & Gini, 2010). Some studies have analyzed the overlap between perpetration and victimization (e.g., bully-victims; Salmivalli & Nieminen, 2002; Veenstra et al., 2005), but research examining the co-occurrence and temporal fluctuation of bystander roles is still limited (Demaray et al., 2021; Gini et al., 2021). Two key processes, moral disengagement and conformity to peer pressure, may help explain why some adolescents maintain defending behaviors while others become passive or complicit in aggression (Álvarez-Turrado et al., 2024; Falla et al., 2022). Yet, their joint influence on changing bystander roles remains largely unexplored (Romera et al., 2024). This study addresses this gap by identifying joint developmental trajectories of bystander behaviors, defending, passive, and pro-bullying, across secondary education, and by examining how moral disengagement and peer pressure contribute to their stability or change over time.

### Bystander Behavior Variability in Secondary Education

Understanding bystander behavior in bullying requires attention to the individual-level processes that shape adolescents’ varied responses across situations, rather than assuming stable and fixed roles. Traditionally, bystanders have been categorized into static roles: defenders (who support the victim or attempt to stop the bullying, either directly or indirectly), passive bystanders (who observe without acting), and pro-bullying bystanders (who support the aggressor either through active encouragement or by silently reinforcing the behavior; Salmivalli et al., 1996). However, recent research increasingly challenges this fixed-role approach, highlighting that adolescents often alternate between diffent types of behaviors depending on the situation, their goals, and the surrounding peer dynamics (Waasdorp & Bradshaw, 2018). In adolescence, peer interactions intensify, concerns about social status increase, and the need for group belonging becomes more pressing, these social dynamics are especially pronounced, making this developmental stage key for studying changes in bystander behavior over time (Laursen & Veenstra, 2021). From a role theory perspective (Biddle, 1986), bystander behavior should not be understood as the expression of fixed personality traits, but rather as the result of individuals responding to situational demands by adopting and performing social roles in ways shaped by their personal histories, values, and beliefs. A student may defend in one situation and remain passive in another, not only due to changing external conditions but also because of their own stable yet versatile behavioral repertoire. These behavioral enactments are consistent with how adolescents make sense of situations through their own lens, guided by internalized expectations and the social demands. This perspective highlights the importance of studying behavioral trajectories, rather than isolated actions, to better understand how adolescents develop patterns of response over time.

Recent person-centered approaches have begun to capture the behavioral complexity involved in bullying dynamics during secondary education. Using latent class analysis with a large sample of adolescents, five distinct bystander profiles were identified, including contributor and inconsistent types, which deviate from traditional categorical models (Waasdorp & Bradshaw, 2018). Another study extended this by showing that defending behaviors not only change in intensity over time but also shift in form, as adolescents move between qualitatively different behavioral profiles (Bravo et al., 2023). These profiles shifts are particularly relevant within secondary education settings, where students are repeatedly exposed to diverse social contexts, across classrooms, school years, and peer groups, that may foster variability in bystander responses. These findings highlight the need to move beyond static classifications and to consider individual variability in bystander responses across time, particularly during adolescence when identity formation and social positioning are in flux (Gini et al., 2021). Other studies have examined broader patterns of engagement among adolescents. For example, in a latent profile analysis, classes such as “Victimized Defenders” and “Bully-Victim-Defenders” were identified, suggesting that adolescents may simultaneously occupy multiple bullying-related roles (Jenkins et al., 2021). Similarly, another study identified profiles characterized by broad behavioral involvement (e.g., bullying, assisting, defending, and victimization) (Demaray et al., 2021). While both studies highlight the complexity and overlap of bullying profiles during adolescence, they did not specifically examine the co-occurrence of different bystander behaviors. Among the limited research examining joint developmental trajectories of bullying participation roles, most studies have focused primarily on victimization and perpetration rather than on bystander behavior. For example, two studies identified developmental trajectories of bullying and victimization across secondary school years, showing that individuals often shift between roles and that such patterns relate to social positioning and behavioral change (de Vries et al., 2021; Zhou et al., 2020). However, neither study focused specifically on bystander behavior. Given the dynamic peer contexts of secondary education, this lack of attention to overlapping bystander roles is a key gap in the literature (Pouwels & Garandeau, 2021).

### Moral Disengagement and Conformity To Peer Pressure in Bystanders’ Behavioral Trajectories

Bystander behavior in bullying situations is shaped by the interaction of personal and social factors, embedded within the wider peer group structure. As adolescents navigate shifting social hierarchies, their position within the peer group becomes increasingly relevant (Salmivalli et al., 2021). Within this social framework, individual cognitive strategies such as moral disengagement (e.g., justifying inaction) and social tendencies such as conformity to peer norms (e.g., susceptibility to peer pressure) have been identified as significant predictors of bystander behaviors in peer aggression contexts (Zhang et al., 2022). These constructs are examined in the present study due to their well-established links to bystander decision-making in cross-sectional research; however, their inclusion also allows us to explore how they may influence behavioral patterns over time (Aisyah et al., 2025). Indeed, it remains crucial to examine whether baseline levels of moral disengagement and peer conformity relate to longitudinal changes in bystander responses, clarifying their role as potential drivers of behavioral trajectories over time and shedding light on potential mechanisms through which bystander roles become consolidated, shift, or even reverse across adolescence.

Moral disengagement refers to a set of psychological mechanisms that enable individuals to disconnect moral standards from unethical behavior, allowing them to act in ways that would typically violate their own values without experiencing self-condemnation (Bandura, 2018). This construct is rooted in Bandura’s social cognitive theory of moral agency (Bandura, 2016), which posits that behavior is guided by a triadic interplay between personal factors, behavioral patterns, and environmental influences. Within this framework, moral standards do not automatically regulate conduct; instead, self-regulatory processes must be activated to align behavior with moral beliefs (Sjögren et al., 2024). In adolescence, moral disengagement has been consistently associated with passive and even reinforcing bystander roles in cross-sectional research (Bjärehed et al., 2020). Furthermore, longitudinal studies show that higher initial levels of moral disengagement predict increased passivity over time, while decreases in moral disengagement across time are associated with a greater likelihood of defending victims (Doramajian & Bukowsky, 2015). Although Bandura’s framework might suggest that moral disengagement decreases as adolescents gain autonomy and consolidate their moral identity (Paciello et al., 2008), particularly in the later stages of secondary education, recent findings indicate a more complex pattern of change over time. Due to the important role of moral disengagement in bystander behavior and the variability of this cognitive mechanism in adolescence, it is expected that changes in moral disengagement are related to changes in bystander trajectories.

In addition to moral disengagement, adolescents´ tendency to conform to peer pressure is thought to play a key role in shaping bystander behavior. Conformity reflects an individual´s inclination to align with peers´ expectations or behaviors, even when these conflict with personal values or moral standards (Palani & Mani, 2016). Drawing from Asch’s paradigm, which demonstrates how individuals often align their judgements with a group´s consensus even when they know the group is wrong (Asch, 1956), this conformity effect has been observed in moral decision-making contexts (Kundu & Cummins, 2013). In such situations, adolescents are especially sensitive to peer influence, often prioritizing social acceptance and the avoidance of rejection over their own independent moral reasoning or adherence to personal values (Farrell et al., 2017).

In bullying scenarios, this susceptibility may translate into reluctance to intervene, not necessarily due to moral disengagement, but to concerns about the potential costs of intervention for their own status (Romera et al., 2019). However, as adolescents progress through secondary education, their orientation toward peer relationships and group belonging changes: while group acceptance remains important, they increasingly balance this with greater personal agency, which may allow them to resist conformity and act according to internalized moral values (Laursen, 2018). Conformity to peer pressure has been shown to significantly predict bystander roles across time: adolescents who score higher on peer conformity are more likely to maintain passive or pro-bullying roles (Pozzoli & Gini, 2021). These findings support the view that susceptibility to peer influence plays a key role in sustaining less prosocial bystander behaviors. However, as adolescents progress through secondary education and their need for group approval evolves, they may become more inclined to prioritize personal values over peer expectations, potentially altering these trajectories over time.

## Current Study

Although bystanders play a crucial role in bullying dynamics, little research has examined how different bystander behaviors co-occur and change over time during adolescence. To address this gap, the present study examined the developmental trajectories of bystander behaviors in bullying throughout secondary school years. The first objective was to examine how defender, passive, and pro-bully bystander behaviors co-evolve during this period. It was expected that distinct longitudinal patterns would emerge, including stable engagement in specific profiles as well as divergent developmental trajectories over time (Hypothesis 1). The second objective was to explore how moral disengagement and conformity to peer pressure are associated with bystander trajectories throughout the secondary school years. It was hypothesized that higher baseline levels of moral disengagement and conformity to peer pressure will be associated with stable or increasing engagement in passive and pro-bullying behaviors, whereas lower baseline levels will be linked to defender trajectories (Hypothesis 2). In addition to examining baseline associations, the longitudinal trajectories of moral disengagement and conformity to peer pressure will also be explored, although no specific directional hypotheses are proposed for these trends. It was expected that distinct longitudinal patterns would emerge, reflecting stable engagement in specific roles as well as divergent developmental trajectories.

## Method

### Participants and Procedure

**Fig. 1:**
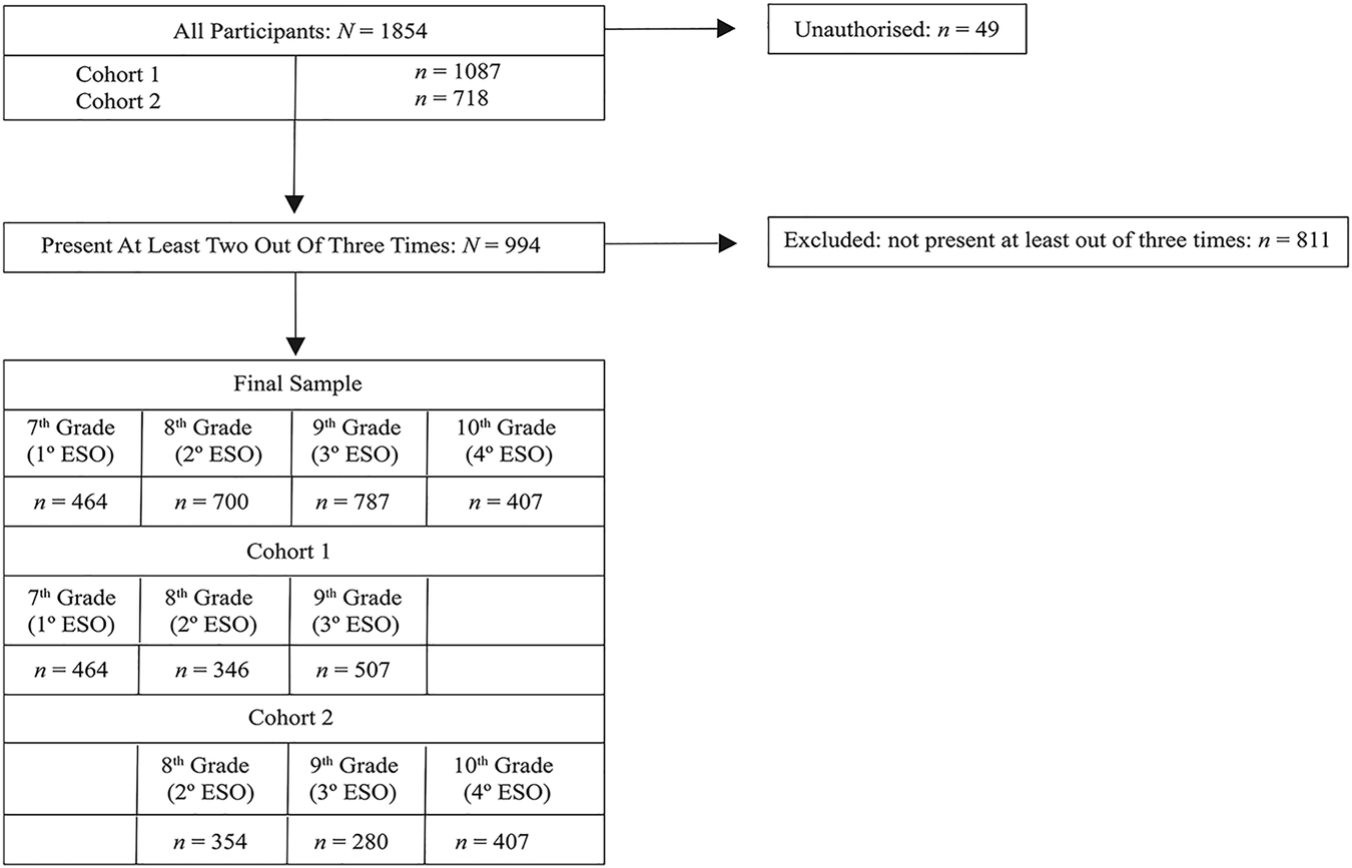
Flowchart of included participants

A flowchart of the included participants is presented in Fig. 1. The initial study sample included 1854 students, aged between 11 and 17 years (48% girls, *M*_age_ = 13.9; *SD* = 0.85), enrolled in 7^th^ Grade (1º ESO in the Spanish educational system) to 10^th^ Grade (4º ESO), across 14 schools in the south of Spain. All schools were subject to the regional *convivencia* framework, which establishes general guidelines for promoting positive coexistence and preventing school violence. A total of 49 students (3%) were not authorized by their families to participate in the study. For this longitudinal study, data were collected in three waves over a two-year period to track changes throughout the secondary education stage (four years in Spain). A 12-month interval elapsed between each wave, allowing for the observation of annual changes and trajectories in the variables of interest. The first wave took place in May 2022 (T1) and involved 984 students. The second wave occurred in May 2023 (T2) with 701 students participating, and the third wave took place in May 2024 (T3) with a total of 1169 students. Attrition was due to school changes or absence during data collection.

To cover the entire four-year secondary education stage with only three waves, two partially overlapping cohorts were followed. Each cohort participated in three data collection waves over two years. Cohort 1 began in 7^th^ Grade (1º ESO) at T1, moved to 8^th^ Grade (2º ESO) at T2, and then to 9^th^ Grade (3º ESO) at T3 (Cohort 1: *n* = 1087). Similarly, Cohort 2 started in 8^th^ Grade (2º ESO) at T1, progressed to 9^th^ Grade (3º ESO) at T2, and finished in 10^th^ Grade (4º ESO) at T3 (Cohort 2: *n* = 718). This design allows us to capture the trajectories across the four academic years, even though each individual provided data on a maximum of three occasions only. To obtain each student’s trajectory, it was necessary for them to have participated in at least two of the three measurement points. This resulted in a sample of 994 adolescents: 556 from Cohort 1 (48% girls, *M*_age_ = 12.5, *SD* = 0.6) and 438 from Cohort 2 (48% girls, *M*_age_ = 13.5, *SD* = 0.6). To analyze whether the participants included and excluded from the study showed significant differences in the study variables, a logistic regression analysis was conducted. The results indicated that students who participated in two or more waves did not significantly differ on any of the study variables from those who participated in only one wave (*p*s > .05).

This cohort-based sampling strategy aligns with a planned missing data design, specifically a wave missing design, which is commonly used in longitudinal research to optimize data collection over time (Little & Rhemtulla, 2013). This approach enables the efficient assessment of behavioral trajectories across all secondary school years (resulting in three waves of data in the present study), despite not collecting data from all students at each wave.

The Monte Carlo simulation developed using *Mplus* was employed to assess the adequacy of the sample size and the statistical power of the analyses conducted. This allowed us to determine whether the sample size was sufficient to detect significant associations and to ensure the stability of the obtained estimates (Nylund et al., 2007). The results indicated that a sample size of 35 students would be sufficient to detect a moderate within-person effect size of 0.20 (Cohen, 1988) with a power of 0.80 and an alpha of 0.05. Considering the minimum threshold of 5% recommended for establishing a profile in this type of study (Nguena et al., 2020), a sample of 994 adolescents would be sufficient to obtain a profile with at least 49 participants, exceeding the 35 recommended as the minimum based on the sample size analysis. This ensures greater robustness in the results, guaranteeing the reliable detection of effects on change trajectories in larger and more heterogeneous samples.

The study was approved by the Bioethics and Biosafety Committee of the University of Cordoba (Spain). Data collection was conducted in educational centers during school hours, where detailed information about the project’s purpose was provided. After obtaining approval from the administrative teams of each school, informed consent was requested from the legal guardians of the students. The questionnaires were administered by trained psychologists and educators with research experience. Special emphasis was placed on the voluntary and confidential nature of data collection. Students were given standardized instructions to complete the questionnaire, which took approximately 40 min. To link surveys over time, students were asked to create their own personal code, ensuring that only they knew this identification, thus maintaining the anonymity of the data.

### Instruments

The following instruments were administered at each of the three measurement points (T1, T2, and T3).

#### Bystander behavior

The bystander response was assessed using the *Student’s Bystander Behaviour Scale* (SBBS) (Thornberg et al., 2017). This scale consists of 15 items that measure bystander behavior in bullying situations and covers the three possible roles they may adopt: passivity, support for the aggressor (pro-bully), and defense of the victim. First, students were asked: “Try to recall situations in which you have witnessed a peer being victimized (e.g., being mocked, threatened, physically attacked, excluded, etc.). What do you usually do?”. Five items assessed pro-bully behavior (e.g., “I also take part in bullying the peer”), five measured passive responses (e.g., “I do not do anything in particular”), and five described defense behaviors (e.g., “I defend the peer who is being bullied”). Responses were given on a 7-point Likert scale (1 = “Strongly disagree” to 7 = “Strongly agree”), reflecting the extent to which students felt the described behaviors aligned with how they typically act when witnessing peer victimization. The scale demonstrated good internal consistency in the analyzed sample (Passive: ω_T1_ = 0.78, ω_T2_ = 0.82, ω_T3_ = 0.82; Pro-bully: ω_T1_ = 0.75, ω_T2_ = 0.82, ω_T3_ = 0.85; Defending: ω_T1_ = 0.79, ω_T2_ = 0.85, ω_T3_ = 0.88). Confirmatory factor analysis (CFA) supported the three-factor structure and indicated good psychometric properties at all three time points: T1 (χ² = 290, *df* = 87, *p* < .001; CFI = 0.955; TLI = 0.945; RMSEA = 0.076, 90% CI [0.066, 0.085]), T2 (χ² = 256, *df* = 87, *p* < .001; CFI = 0.970; TLI = 0.964; RMSEA = 0.079, 90% CI [0.068, 0.090]), and T3 (χ² = 396, *df* = 87, *p* < .001; CFI = 0.962; TLI = 0.955; RMSEA = 0.088, 90% CI [0.079, 0.097]).

#### Moral disengagement

Moral disengagement was measured using *The Moral Disengagement Scale for Adolescents* (MDS-24) (Caprara et al., 1996), adapted into Spanish (Romera et al., 2023). This questionnaire includes 24 items that assess moral disengagement, with responses recorded on a five-point Likert scale ranging from 1 = “Strongly disagree” to 5 = “Strongly agree”. The internal consistency was excellent at each timepoint (ω_T1_ = 0.85, ω_T2_ = 0.90, ω_T3_ = 0.91). CFA supported the expected unidimensional structure, showing acceptable to good fit: T1 (χ² = 1006, *df* = 252, *p* < .001; CFI = 0.894; TLI = 0.884; RMSEA = 0.079, 90% CI [0.074, 0.084]), T2 (χ² = 881, *df* = 252, *p* < .001; CFI = 0.957; TLI = 0.953; RMSEA = 0.076, 90% CI [0.070, 0.081]), and T3 (χ² = 995, *df* = 252, *p* < .001; CFI = 0.898; TLI = 0.888; RMSEA = 0.082, 90% CI [0.077, 0.088]).

#### Conformity to peer pressure

Conformity to peer pressure was assessed using the 11-item *Peer Pressure Scale* (Santor et al., 2000). This scale measures the subjective experience of feeling vulnerable to peer pressure, including both risk behaviors and non-antisocial situations. The items included statements such as “I often feel pressured to do things I wouldn’t normally do” or “I have skipped class when others convinced me to do so.” Responses are rated on a five-point Likert scale, ranging from 1 = “Strongly disagree” to 5 = “Strongly agree”. The scale demonstrated good internal consistency across all three time points (ω_T1_ = 0.75, ω_T2_ = 0.88, ω_T3_ = 0.85). CFA supported the unidimensional structure, with acceptable model fit: T1 (χ² = 288, *df* = 44, *p* < .001; CFI = 0.889; TLI = 0.861; RMSEA = 0.115, 90% CI [0.102, 0.127]), T2 (χ² = 242, *df* = 44, *p* < .001; CFI = 0.959; TLI = 0.949; RMSEA = 0.101, 90% CI [0.089, 0.114]), and T3 (χ² = 227, *df* = 44, *p* < .001; CFI = 0.957; TLI = 0.947; RMSEA = 0.096, 90% CI [0.084, 0.108]).

### Data Analysis

Descriptive statistics and bivariate correlations among the study variables were computed for each data collection wave (T1–T3) to examine distributions, interrelationships, and multicollinearity. These analyses provided an overview of the distinctiveness and baseline associations among the socio-moral variables. To address the objectives of this study, two main steps were carried out using *Mplus* software version 8.11 (Muthén & Muthén, 1998–2017). In the first step, the longitudinal trajectories of bystander responses to bullying (passive, pro-bullying, and defending) across the four school years, from 7^th^ Grade (equivalent to 1º ESO) to 10^th^ Grade (4º ESO), were identified. This was conducted through a parallel-process Latent Class Growth Analysis (LCGA) to capture the joint trajectories of passive, pro-bully, and defense responses among bystanders. This approach extended the latent class growth analysis model to a parallel process model, allowing multiple growth trajectories to be examined simultaneously (Cruz et al., 2017). The trajectory indicators used were the intercept (initial levels) and the slope (evolution over time). Three models (ranging from one to six classes) were estimated. Multiple fit indices were used to determine the optimal number of trajectory classes. First, the Akaike Information Criterion (AIC), the Bayesian Information Criterion (BIC), and the sample-size adjusted BIC (a-BIC) were considered. Models with lower AIC, BIC, and a-BIC values were considered better solutions. Additionally, the Vuong-Lo-Mendell-Rubin Likelihood Ratio Test (VLMR-LRT) and the adjusted Lo-Mendell-Rubin Likelihood Ratio Test (LMR-LRT) compared the model with *k* trajectory classes against a model with *k − 1* trajectory classes. A significant *p*-value in the VLMR-LRT and adjusted LMR-LRT indicated that a model with *k* trajectory classes provided a better fit than a model with *k − 1* trajectory classes. Furthermore, the entropy value was used to assess classification accuracy, ranging from 0 to 1, with values closer to 1 indicating higher classification precision. To maximize the generalizability of the results, it was established that each class size should represent at least 5% of the total sample (Nguena et al., 2020). In addition to fit indices, the substantive interpretability of the trajectory classes was evaluated, considering their conceptual relevance. Little’s Missing Completely at Random (MCAR) Test (Little, 2013) was conducted to evaluate the missing data mechanism. Results were significant at each wave (T1: χ²/*df* = 1.25, *p* < .001, T2: χ²/*df* = 1.30, *p* < .001, T3: χ²/*df* = 1.45, *p* < .001), indicating that the data were not strictly MCAR. However, the fact that χ²/*df* ratios remained well below the conventional threshold of 5, the missingness pattern was considered consistent with the less restrictive assumption of *Missing at Random* (MAR) (Bollen, 1989). Under this assumption, the use of *Full Information Maximum Likelihood* (FIML) is appropriate and yields unbiased estimates. Accordingly, model estimation was performed using robust maximum likelihood (MLR), and missing data were handled with FIML, ensuring that all available information contributed to the analyses without listwise deletion or imputation. To enhance transparency, the extent of item-level missingness (Table S1) and group comparisons by number of waves completed (Table S2) are reported in the Online Resource.

Subsequently, chi-square difference tests were conducted to evaluate associations between gender and membership in the different trajectory classes. The effect size of the differences was assessed using the odds ratio (OR), categorized as negligible (OR < 1.68), small (1.68 ≤ OR < 3.47), moderate (3.47 ≤ OR < 6.71), and large (OR ≥ 6.71) (Chen et al., 2010).

In the second step, a parallel- process LCGA was conducted to examine the longitudinal trajectories of moral disengagement and conformity to peer pressure. Independent growth curves for each construct were included in the model to assess how the identified bystander response profiles relate to individual patterns of change in moral disengagement and conformity to peer pressure over time. Differences in intercepts and slopes between trajectories were tested for statistical significance using the Wald test (Muthén & Muthén, 1998–2017).

## Results

### Preliminary Analyses

Descriptive statistics for all study variables are presented in Table 1. Bivariate correlations are reported in Table 2. As expected, defending correlated negatively with pro-bullying behavior, moral disengagement and conformity to peer pressure, whereas pro-bullying and passive behaviors were positively related to both socio-moral variables.

**Table 1:** Descriptive statistics

Variables	*n*	M (SD)	Skewness	Kurtosis
1. Pro-bully bystander behavior (T1)	729	1.31 (0.65)	3.05	11.23
2. Pro-bully bystander behavior (T2)	559	1.41 (0.86)	2.80	8.88
3. Pro-bully bystander behavior (T3)	865	1.29 (0.69)	3.34	13.42
4. Passive bystander behavior (T1)	707	2.75 (1.27)	0.62	0.15
5. Passive bystander behavior (T2)	565	2.90 (1.41)	0.49	-0.48
6. Passive bystander behavior (T3)	844	3.11 (1.35)	0.28	-0.52
7. Defender bystander behavior (T1)	734	5.11 (1.40)	-0.77	0.15
8. Defender bystander behavior (T2)	561	4.85 (1.54)	-0.55	-0.38
9. Defender bystander behavior (T3)	849	4.44 (1.56)	-0.34	-0.55
10. Moral disengagement (T1)	690	1.76 (0.56)	1.81	4.82
11. Moral disengagement (T2)	666	1.56 (0.60)	1.73	3.59
12. Moral disengagement (T3)	804	1.57 (0.56)	1.82	5.04
13. Conformity to peer pressure (T1)	792	1.50 (0.52)	1.67	4.01
14. Conformity to peer pressure (T2)	854	1.48 (0.62)	1.91	3.67
15. Conformity to peer pressure (T3)	876	1.47 (0.59)	1.99	4.84

**Table 2:** Bivariate correlations

Variables	1	2	3	4	5	6	7	8	9	10	11	12	13	14
1. Pro-bully bystander behavior (T1)	-													
2. Pro-bully bystander behavior (T2)	0.29***	-												
3. Pro-bully bystander behavior (T3)	0.22***	0.28***	-											
4. Passive bystander behavior (T1)	0.21***	0.12*	0.13**	-										
5. Passive bystander behavior (T2)	0.18***	0.31***	0.11*	0.47***	-									
6. Passive bystander behavior (T3)	0.08*	0.09	0.19***	0.28***	0.48***	-								
7. Defender bystander behavior (T1)	− 0.23***	− 0.11*	− 0.14***	− 0.29***	− 0.30***	− 0.18***	-							
8. Defender bystander behavior (T2)	− 0.23***	− 0.19***	− 0.10*	− 0.28***	− 0.28***	− 0.27***	0.48***	-						
9. Defender bystander behavior (T3)	− 0.16***	− 0.13**	− 0.17***	− 0.28***	− 0.29**	− 0.23***	0.37***	0.48***	-					
10. Moral disengagement (T1)	0.18***	0.21***	0.18***	0.12**	0.19***	0.09*	− 0.18***	− 0.20***	− 0.12**	-				
11. Moral disengagement (T2)	0.27***	0.47***	0.25***	0.07	0.27***	0.08*	− 0.13**	− 0.13*	− 0.12**	0.34***	-			
12. Moral disengagement (T3)	0.26***	0.26***	0.25***	0.13**	0.22***	0.14***	− 0.09*	− 0.22***	− 0.15***	0.31***	0.50***	-		
13. Conformity to peer pressure (T1)	0.28***	0.32***	0.17***	0.06	0.16***	0.06	− 0.15***	− 0.16***	− 0.12**	0.33***	0.28***	0.27***	-	
14. Conformity to peer pressure (T2)	0.22***	0.32***	0.20***	− 0.01	0.19***	0.07	− 0.07	− 0.21***	− 0.11**	0.21***	0.38***	0.31***	0.30***	-
15. Conformity to peer pressure (T3)	0.25***	0.26***	0.21***	0.01	0.20***	0.11**	− 0.04	− 0.15**	− 0.17***	0.16***	0.29***	0.37***	0.30***	0.45***

Note. *p < .05. **p < .01. ***p < .001

### Trajectories of Bystander Responses

To identify the trajectory classes based on bystander responses, models ranging from one to six classes were compared. Table 3 presents the model fit indices for estimating bystanders’ response trajectories using the parallel- process LCGA. Statistical indices (AIC, BIC, and aBIC) indicated that model fit improved as the number of classes increased. However, the results of the likelihood ratio tests (VLMR-LRT and adjusted LMR-LRT) showed that the two-class model was the last to reach statistical significance at *p* < .05. From the three-class model onward, these tests were no longer significant, although *p*-values remained relatively small, suggesting that models with a higher number of classes captured some complexity in the data. Additionally, entropy slightly decreased as the number of classes increased but remained above 0.80. Based on these results, the two-class model initially appeared preferable from a statistical perspective. However, a conceptual and theoretical review of the model revealed that a two-class solution did not adequately represent the expected heterogeneity in bystanders’ response trajectories. The three-class model provided a balance between strong statistical fit and substantive interpretability of the trajectories and was therefore chosen. Moreover, each of the identified classes in this model represented at least 5% of the total sample.

**Table 3:** Model fit indices of parallel process growth mixture models

Number of profiles						AIC	BIC	aBIC	Entropy	VLMR LRT	Adj. LMR LRT
1. *N* = 994 I_P_ = 2.66*** S_P_ = 0.18*** I_PB_ = 1.33*** S_PB_ = 0.01 I_D_ = 5.23*** S_D_ = -0.31***						19014.68	19205.85	19081.99	-	-	-
2. *N* = 85 (9%) I_P_ = 3.45*** S_P_ = 0.08 I_PB_ = 2.59*** S_PB_ = 0.15 I_D_ = 4.62*** S_D_ = -0.28*	*N* = 909 (91%) I_P_ = 2.59*** S_P_ = 0.19*** I_PB_ = 1.21*** S_PB_ = -0.02 I_D_ = 5.29*** S_D_ = -0.31***					18487.47	18712.95	18566.85	0.96	< 0.05	< 0.05
3. ***N* = 67 (7%)** **I**_**P**_ = 2.98*** **S**_**P**_ = 0.33** **I**_**PB**_ = 1.48*** **S**_**PB**_ = 0.72*** **I**_**D**_ = 5.10*** **S**_**D**_ = -0.49***	***N*** **= 875 (88%)** **I**_**P**_ = 2.58*** **S**_**P**_ = 0.18*** **I**_**PB**_ = 1.18*** **S**_**PB**_ **= -0.02** **I**_**D**_ = 5.31*** **S**_**D**_ = -0.31***	***N*** **= 52 (5%)** **I**_**P**_ = 3.68*** **S**_**P**_ **= -0.15** **I**_**PB**_ = 3.73*** **S**_**PB**_ = -0.80*** **I**_**D**_ = 3.97*** **S**_**D**_ **= 0.03**				**18188.98**	**18448.77**	**18280.44**	**0.93**	**0.10**	**0.11**
4. *N* = 857 (86%) I_P_ = 2.58*** S_P_ = 0.18*** I_PB_ = 1.19*** S_PB_ = -0.03** I_D_ = 5.30*** S_D_ = -0.30***	*N* = 57 (6%) I_P_ = 3.74*** S_P_ = -0.17 I_PB_ = 3.60*** S_PB_ = -0.68*** I_D_ = 4.19*** S_D_ = -0.10	*N* = 28 (3%) I_P_ = 3.77*** S_P_ = 0.16 I_PB_ = 1.89*** S_PB_ = 0.82*** I_D_ = 4.74*** S_D_ = -0.26	*N* = 52 (5%) I_P_ = 2.36*** S_P_ = 0.51*** I_PB_ = 1.13*** S_PB_ = 0.64*** I_D_ = 5.40*** S_D_ = -0.66***			18039.74	18333.84	18143.28	0.94	0.30	0.31
5. *N* = 6 (1%) I_P_ = 3.59*** S_P_ = 0.93* I_PB_ = 2.18*** S_PB_ = 1.60*** I_D_ = 2.46*** S_D_ = 1.36***	*N* = 776 (78%) I_P_ = 2.56*** S_P_ = 0.21*** I_PB_ = 1.08*** S_PB_ = 0.02* I_D_ = 5.37*** S_D_ = -0.26***	*N* = 46 (5%) I_P_ = 3.66*** S_P_ = -0.12 I_PB_ = 3.91*** S_PB_ = -0.84*** I_D_ = 3.91*** S_D_ = 0.12	*N* = 104 (10%) I_P_ = 2.98*** S_P_ = -0.11 I_PB_ = 2.13*** S_PB_ = -0.37*** I_D_ = 4.79*** S_D_ = -0.78***	*N* = 62 (6%) I_P_ = 2.71*** S_P_ = 0.40** I_PB_ = 1.20*** S_PB_ = 0.77*** I_D_ = 5.34*** S_D_ = -0.61***		17894.68	18223.10	18010.31	0.90	0.36	0.36
6. *N* = 5 (1%) I_P_ = 3.79*** S_P_ = 0.86* I_PB_ = 2.09*** S_PB_ = 1.59*** I_D_ = 2.52*** S_D_ = 1.28***	*N* = 32 (3%) I_P_ = 3.68*** S_P_ = -0.20 I_PB_ = 4.16*** S_PB_ = -1.04*** I_D_ = 3.91*** S_D_ = 0.33	*N* = 98 (10%) I_P_ = 3.10*** S_P_ = 0.16 I_PB_ = 2.22*** S_PB_ = 0.90* I_D_ = 4.64*** S_D_ = -0.43***	*N* = 717 (72%) I_P_ = 2.51*** S_P_ = 0.23*** I_PB_ = 1.11*** S_PB_ = -0.01 I_D_ = 5.41*** S_D_ = -0.20***	*N* = 92 (9%) I_P_ = 3.05*** S_P_ = -0.26 I_PB_ = 1.14*** S_PB_ = -0.02 I_D_ = 4.87*** S_D_ = -1.08**	*N* = 50 (5%) I_P_ = 2.51*** S_P_ = 0.45*** I_PB_ = 1.14*** S_PB_ = 0.87*** I_D_ = 5.64*** S_D_ = -0.70***	17776.40	18139.12	17904.10	0.87	0.22	0.22

*N* = number of students in class based on most likely latent class memberships; *I* = intercept; *S* = slope; *P* = passive; *PB* = pro-bully; *D* = defender; *AIC* = Akaike Information Criterion; *BIC* = Bayesian Information Criterion; *aBIC* = adjusted Bayesian Information Criterion; *VLMR LRT* = Vuong-Lo-Mendell-Rubin likelihood ratio test; *Adj. LMR LRT* = adjusted Lo–Mendell–Rubin likelihood ratio test. Selected model is highlighted in bold.

*p < .05. **p < .01. ***p < .001.

**Fig. 2:**
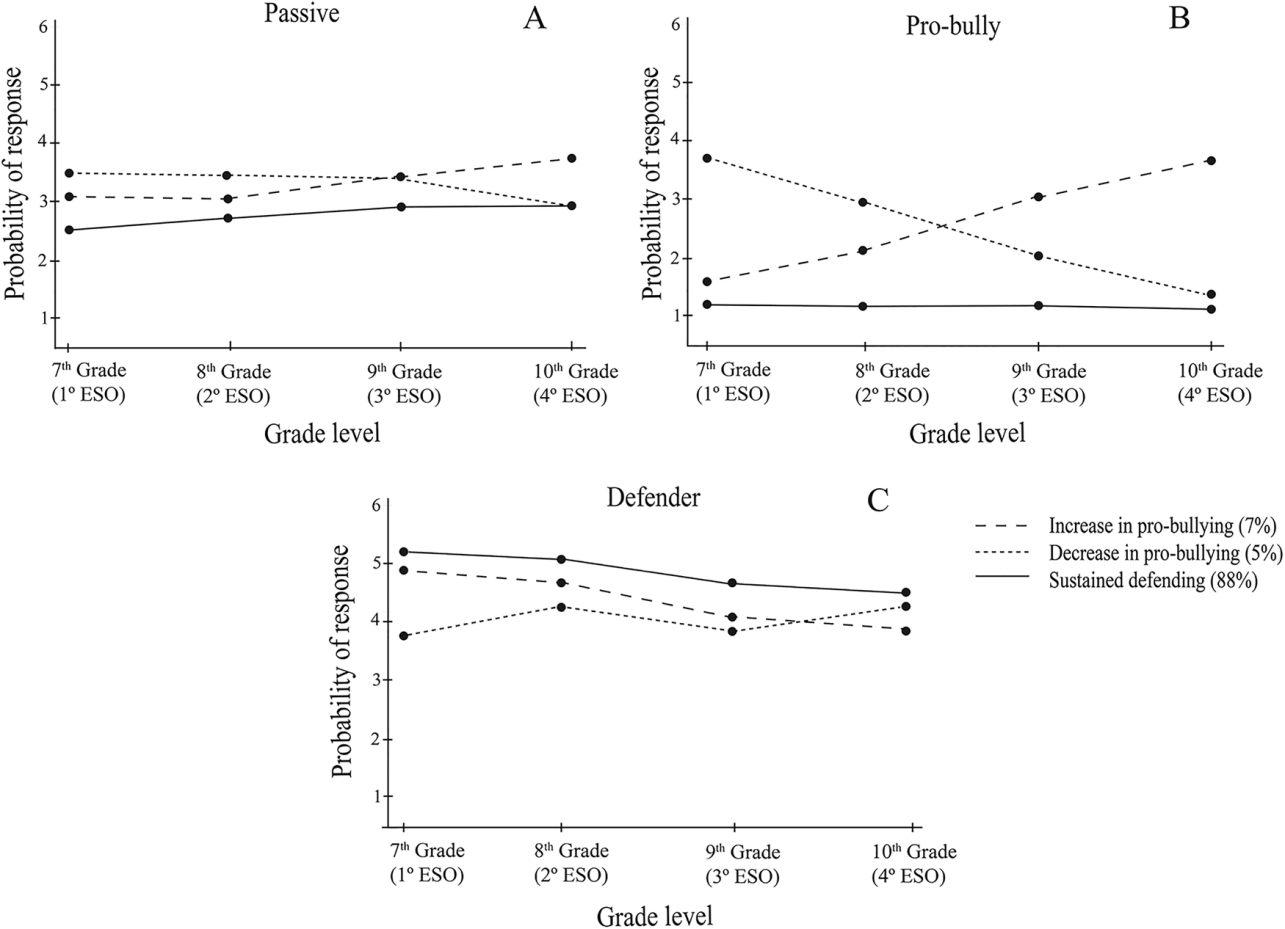
Joint trajectories classes of bystander behaviors: (a) Passive, (b) Pro-bully and (c) Defender

Figure 2 presents the baseline class probabilities and the longitudinal trajectories of the three bystander response profiles. Each panel corresponded to a baseline profile (intercept/initial level) and illustrates how adolescents’ probabilities of defending, passivity, and pro-bullying behaviors evolved across waves. Adolescents in Profile 1 were labelled as *Increase Pro-bullying* (7%). This group was characterized by initially high levels of defending and passivity, and low pro-bullying. However, over time, they showed a significant decrease in their defensive response and a notable increase in both pro-bullying and passive behaviors. Profile 2 was characterized by high levels of defending, low pro-bullying, and low passivity, leading to their classification as *Sustained Defending* (88%). Finally, Profile 3 was labelled as *Decrease Pro-bullying* (5%). Adolescents in this group initially exhibited high levels of passivity and pro-bullying, and low defending behavior. However, over time, they transitioned towards more defensive responses, reducing their pro-bullying behaviors while maintaining their relatively high level of passivity.

Chi-square tests showed significant gender effects. Being a boy significantly increased the likelihood of being classified in the *Increase Pro-bullying* profile (OR = 2.99, 95% CI [1.51; 5.91]) and the *Decrease Pro-bullying* profile (OR = 2.73, 95% CI [1.15; 6.47]). Girls were more likely to belong to the *Sustained Defending* profile.

### Joint Trajectories and their Co-evolution with Moral Disengagement and Conformity To Peer Pressure

Next, the three trajectories were examined in relation to adolescents’ moral disengagement and conformity to peer pressure during their secondary education years. Table 4 presents the differences between the three profiles in the intercepts in 7^th^ Grade (1º ESO) and 10^th^ Grade (4º ESO) of secondary education, as well as differences in their progression.

**Table 4:** Model fit indices for moral disengagement and conformity to peer pressure trajectories: three class solution

	Trajectory 1: Increase Pro-bullying (*N* = 67)	Trajectory 2: Decrease Pro-bullying (*N* = 52)	Trajectory 3: Sustained Defending (*N* = 875)	AIC	BIC	aBIC	Entropy
Moral disengagement	I_7_= 2.02***(1–3) S = -0.01 I_10_ = 1.98*** (1–3)	I_7_ = 2.50***(2–3) S = -0.20 I_10_ = 1.90***	I_7_ = 1.68***(1–3, 2–3) S = -0.06*** I_10_ = 1.50***(1–3)	22811.203	23193.539	22945.807	0.940
Conformity to peer pressure	I_7_ = 1.61*** S = 0.13* I_10_ = 1.99***(1–3)	I_7_ = 2.13***(2–3) S = -0.08 I_10_ = 1.89***	I_7_ = 1.49***(2–3) S = -0.03* I_10_ = 1.40***(1–3)	22453.760	22836.096	22588.364	0.936

*N* = number of students in class based on most likely latent class memberships; I = intercept; S = slope; 7 = 7^th^ Grade (1º ESO); 10 = 10^th^ Grade (4º ESO) (I_10_ has been estimated in a different model); the differences between trajectories are shown in brackets

*p < .05. **p < .01. ***p < .001

**Fig. 3:**
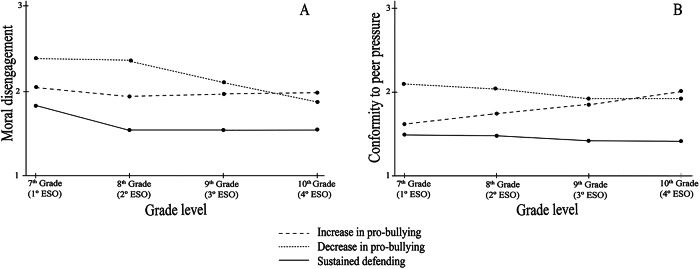
Changes in forms of bystander behaviors over time by trajectory class: (a) Moral disengagement and (b) Conformity to peer pressure

The results of the Wald test indicated that, at the 7^th^ Grade time point, students in the *Increase Pro-bullying* profile exhibited a significantly higher initial level of moral disengagement compared to the *Sustained Defending* profile (χ²_(1)_ = 5.82, *p* < .05, *d* = 0.34) (Table 2, see also Fig. 3a). Over time, both profiles followed a downward trajectory in moral disengagement (Table 2; Fig. 3), with the difference between them remaining significant by the 10^th^ Grade (χ²_(1)_ = 9.68, *p* < .01, *d* = 0.49).

In 7^th^ Grade, adolescents in the *Decrease Pro-bullying* profile showed a higher initial level of moral disengagement compared to those in the *Sustained Defending* profile, with a large effect size (χ²_(1)_ = 11.72, *p* < .001, *d* = 0.82). However, despite the high initial levels, their trajectory declined over time (see Table 4). As a result of these changes, the difference between the two profiles was no longer significant by the 10^th^ Grade. No differences in moral disengagement were found between the *Increase Pro-bullying* and *Decrease Pro-bullying* profiles, either in 7^th^ Grade or in their temporal progression.

Regarding conformity to peer pressure, the results of the Wald test showed that at the 7^th^ Grade time point, there were no significant differences between adolescents in the *Increase Pro-bullying* and *Sustained Defending* profiles (see Fig. 3b). However, over time, the *Increase Pro-bullying* profile followed an upward trajectory, whereas the *Sustained Defending* profile showed a declining trend (see Table 4). As a result, by the 10^th^ Grade, the difference between the two profiles was significant, with the *Increase Pro-bullying* group exhibiting higher levels of conformity to peer pressure, with a moderate effect size (χ²_(1)_ = 14.11, *p* < .001, *d* = 0.59). On the other hand, students in the *Decrease Pro-bullying* profile initially showed higher peer pressure levels compared to those in the *Sustained Defending* profile, with a moderate effect size (χ²_(1)_ = 3.98, *p* < .05, *d* = 0.64). Over time, both profiles followed a downward trajectory (see Table 4), and the initial difference was no longer significant by the 10^th^ Grade. No differences in peer pressure were found between the *Increase Pro-bullying* and *Decrease Pro-bullying* profiles, either in 7th Grade or in their temporal progression.

## Discussion

Research on bystander behavior in bullying has traditionally conceptualized defending, passive, and pro-bullying roles as static and mutually exclusive categories. However, emerging evidence indicates that adolescents may shift between roles over time, reflecting developmental changes and the interplay of moral and relational dynamics, relational dynamics. Despite this, little is known about how bystander behaviors evolve in tandem or about the socio moral mechanisms that underlie changes across different bystander behaviors. Addressing this gap requires a developmental perspective that integrates both intrapersonal and interpersonal factors shaping behavioral change. This study contributes to this effort by showing that bystander behavior unfolds along distinct developmental trajectories shaped by socio-moral processes, underscoring the need to view it as a dynamic phenomenon rather than as fixed role categories.

The first objective was to examine whether adolescents follow distinct and heterogeneous joint trajectories in defender, passive, and pro-bully behaviors during secondary school. Based on existing literature and the Role Theory (Thornberg et al., 2022), Hypothesis 1 proposed the emergence of multiple behavioral profiles, including both stable and shifting patterns in bystander responses. This hypothesis was supported: using parallel-process LCGA, three distinct profiles were identified: (1) a large group showing sustained defending behavior (*Sustained Defending*), (2) a smaller group with increasing pro-bullying and decreasing defending (*Increase Pro-bullying*), and (3) another small group with the opposite pattern, decreasing pro-bullying and increasing defending (*Decrease Pro-bullying*). These results align with recent person-centered studies suggesting that adolescents do not consistently adhere to fixed roles but may adapt their responses as they interpret and negotiate their social environments (Waasdorp & Bradshaw, 2018; Bravo et al., 2023). Notably, the absence of profiles showing simultaneous increases in defending and passivity, or in pro-bullying and defending, suggests that these behaviors may not typically co-occur over time. While hybrid patterns have been proposed in cross-sectional studies (e.g., Camodeca & Nava, 2022), the current longitudinal data suggest that adolescents tend to consolidate either prosocial or antisocial tendencies over time. Although these additional patterns provide valuable insights into the variability of bystander responses, it is important to note that they represent a relatively small proportion of adolescents. Therefore, caution is warranted when generalizing these trajectories, as they may reflect minority pathways rather than normative developmental trends.

The *Sustained Defending* profile, comprising the majority of adolescents (88%), was characterized by consistently high levels of defending and low involvement in passive or pro-bullying behaviors. This pattern suggests a relatively stable form of prosocial engagement (Bravo et al., 2023). However, a slight decrease in defending and increase in passivity were observed at the end of secondary education, particularly between 9^th^ and 10^th^ Grade. This finding highlights a specific developmental stage, middle adolescence, as a turning point, where contextual factors such as increased academic pressure, shifting peer norms, and emerging social expectations may weaken the outward expression of defending. In this case, the decline in defending co-occurred with a rise in passivity, suggesting that contextual pressures may inhibit rather than replace prosocial intentions. Notably, this shift occurred even among students assessed within the same data collection wave, suggesting that it reflects contextual or educational stage-related changes rather than a longitudinal developmental trend. In line with Role Theory (Biddle, 1986), these shifts may reflect adaptations in adolescents’ social roles within evolving peer contexts, rather than developmental changes per se. As students’ progress to the final year of secondary education, they may face new social expectations, increase academic pressure, or shifting group dynamics that lead them to reassess the perceived value or risks of intervening in bullying situations. While the inclination to defend may persist, its outward expression can diminish if defending is seen as socially costly, less effective, or misaligned with emerging group norms (Pouwels et al., 2019).

The second profile (7%), marked by increasing pro-bullying behavior and declining defending, presents a potentially concerning pathway. This trajectory also involved growing passivity, suggesting that for some adolescents, passivity may serve as a transitional stance, initially motivated by reluctance or avoidance, and later evolving into more active reinforcement of bullying. The findings indicate that this progression unfolded gradually across study waves and was facilitated by initially high levels of moral disengagement, which were sustained over time, and a subsequent increase in conformity to peer norms. In this case, moral disengagement preceded behavioral change by lowering moral self-sanctions, while rising conformity co-occurred with the consolidation of pro-bullying responses. Importantly, these changes should not be interpreted as the result of normative developmental processes, as the vast majority of students (88%) consistently maintained a defending role, reflecting a stable pattern of prosocial behavior. Instead, the shifts observed in a small subset may represent a divergence from this pattern, potentially driven by contextual pressures and a misalignment in moral reasoning, whereby personal or social gain is prioritized over the well-being of others. This supports the idea that bystander roles are flexible, socially negotiated positions rather than fixed outcomes of maturational change.

Conversely, the third profile (5%) represented a more adaptive shift: declining pro-bullying and passivity and increasing defending. Rather than being interpreted as linear developmental progress, this can be understood as a process of behavioral reorientation, in which adolescents adjust their roles in response to new experiences, revised self-perceptions, or moral reflections (Zhou et al., 2020). Here, the decline in pro-bullying and passivity was evident from the first wave onward, and it was accompanied by a steady reduction in both moral disengagement and peer conformity. The co-occurrence of these declines suggests that changes in socio-moral mechanisms and behavioral reorientation evolved in tandem, reinforcing the role of socio-cognitive adjustment in enabling prosocial trajectories. These findings reinforce the idea that bystander behavior should be understood as a form of negotiation embedded in relational contexts, rather than as a static trait or the outcome of a universal developmental trajectory. Across profiles, passivity deserves particular attention. It emerged as an important and ambivalent position, sometimes reflecting a retreat from active defending, and other times functioning as a steppingstone toward either prosocial or antisocial involvement. Empirically, passivity preceded the escalation into pro-bullying in the *Increase Pro-bullying* profile, whereas in the *Decrease Pro-bullying* profile, it co-occurred with a decline in pro-bullying and an increase in defending. This temporal sequencing supports its dual role as both a risk and protective factor, depending on the socio-moral context. This supports prior research indicating that passivity is not a homogeneous construct but rather a multifaceted behavioral stance influenced by varied emotional, cognitive, and contextual factors (Lambe et al., 2019). For some adolescents, passivity may stem from fear of negative evaluation, moral conflict, or lack of self-efficacy; for others, it may reflect deliberate disengagement or even alignment with aggressive peer norms (Sjögren et al., 2020). Interpreting passivity through this lens underscores its importance as a potential leverage point for intervention. Gender differences were also observed, with boys more likely than girls to belong to both shifting profiles (*Increase* and *Decrease Pro-bullying*). This finding is consistent with literature on gendered patterns in bullying perpetration and bystander behavior (Parris et al., 2020), and may reflect stronger social reinforcement of dominance behaviors among boys. Alternatively, it could point to greater behavioral fluidity among boys in response to peer norms and group positioning.

Regarding the psychosocial mechanisms associated with different behavioral profiles, the present study provides valuable insights into how moral disengagement and conformity to peer pressure are linked to distinct patterns of bystander behavior over time (Objective 2). The findings suggest that these mechanisms exhibit dynamic patterns of change, which may be differentially associated with the evolution of bystander behavior depending on the behavioral trajectory. Consistent with prior research (Gini et al., 2014; Thornberg et al., 2017), adolescents in the *Increase Pro-bullying* (7%) profile exhibited significantly higher initial levels of moral disengagement than their *Sustained Defending* (88%) peers, and this gap persisted over time. These findings align with Bandura´s Social Cognitive Theory of Moral Agency (Bandura, 2016), which posits that self-regulatory processes can be deactivated through moral disengagement, allowing adolescents to distance themselves from its moral criteria. In contrast, the *Decrease Pro-bullying* (5%) group also began with elevated levels of moral disengagement, yet this diminished steadily over time, eventually reaching levels comparable to those in the *Sustained Defending* group. This trajectory supports Hypothesis 2, suggesting that sustained or increasing pro-bullying and passive behaviors are associated with high levels of moral disengagement, whereas its reduction aligns with a shift toward more prosocial roles (Kollerová et al., 2014). While causality cannot be inferred, the observed alignment between changes in behavior and moral disengagement levels points to a potential co-evolution of these processes across different behavioral patterns.

Importantly, conformity to peer pressure followed a more differentiated course. Although no significant differences were observed at the beginning between the *Increase Pro-bullying* (7%) and *Sustained Defending* (88%) profiles, their trajectories diverged markedly over time. Adolescents in the *Increase Pro-bullying* profile exhibited a growing susceptibility to peer influence, suggesting that social conformity becomes increasingly central to sustaining pro-bullying behavior when aggression is normatively reinforced within peer groups (Brechwald & Prinstein, 2011). In this case, increases in conformity preceded and then co-occurred with shifts in behavior, indicating that peer influence can act as both a trigger and a maintaining factor in pro-bullying trajectories. This trajectory is consistent with extensions of Asch’s conformity paradigm to moral decision-making (Kundu & Cummins, 2013), showing that individuals may set aside their own moral standards to align with the group when non-intervention or reinforcement of aggression becomes the norm. Meanwhile, the *Sustained Defending* profile showed a small but consistent decrease in conformity over the last academic years, which may reflect increasing autonomy, confidence in moral reasoning, or a lower need for peer approval during adolescence (Yang et al., 2022).

The *Decrease Pro-bullying* (5%) group further illustrates this dynamic. Although these adolescents started with higher levels of peer pressure conformity, this declined significantly over time, paralleling the reduction in their pro-bullying behaviors. Thus, their behavioral change may be more closely associated with reductions in moral disengagement than with shifts in conformity. This nuance suggests that while peer pressure can contribute to the maintenance of aggressive behavior, its reduction may not be a necessary precondition for disengaging from such roles. Instead, adolescents might begin to adopt more prosocial behaviors even in the presence of some degree of social susceptibility, perhaps when internalized moral values or contextual factors (e.g., school climate, adult support) begin to weigh more heavily in their decision-making (Meter & Card, 2016). Overall, these patterns align with the broader idea that socio-cognitive and social-relational mechanisms jointly shape bystander behavior, but also highlight the importance of disentangling their relative influence across different trajectories. Future studies should further explore the interplay between individual self-regulation processes and peer dynamics to clarify how and when adolescents shift away from harmful bystander roles.

Taken together, these patterns highlight that moral disengagement and peer conformity are differentially associated with distinct bystander profiles. The *Increase Pro-bullying* profile appears to be maintained by a combination of persistent cognitive rationalizations and heightened peer influence. In contrast, the *Sustained Defending* and *Decrease Pro-bullying* profiles may reflect a combination of low or declining moral disengagement and relatively stable or decreasing conformity. These findings suggest that while a majority of students show stable bystander behavior across the secondary school years, a meaningful subset displays variability that may reflect differences in individual cognition and social responsiveness (Meter & Card, 2016). Interventions aimed at reducing harmful bystander behavior should consider targeting both moral reasoning and susceptibility to peer influence, particularly among students showing less consistent patterns. In fact, several existing programs have begun to incorporate these components: for example, the MoralMe program (Romera et al., 2024) explicitly addresses complex moral processes such as disengagement and conformity within a whole-school framework, while other initiatives (e.g., KiVa, Bullying Literature Project- Moral Disengagement Version (BLP-MD) include moral reasoning or socio-emotional training as supporting elements. However, in many cases, these constructs remain peripheral rather than systematically integrated. The findings therefore underscore the need for interventions that place greater emphasis on fostering moral engagement while equipping adolescents with strategies to resist negative peer norms, particularly during transitional school periods when susceptibility to peer influence tends to peak.

Nonetheless, these insights should be interpreted in light of certain limitations. First, all measures were based on self-reports, which may be prone to social desirability and self-perception biases. This is likely to be reflected in the high proportion of individuals belonging to *Sustained Defending* group. Although self-reports are suitable for capturing internal states, future studies should include peer or teacher assessments to validate behavioral data. Second, while the number of measurement waves is adequate for identifying behavioral patterns, the relatively long intervals between waves may have limited the ability to detect more rapid or short-term changes in behavior. More frequent assessments across key developmental stages would help capture critical transitions. Third, the absence of dyadic data (e.g., who defends whom or who follows whom) limits the ability to disentangle individual dispositions from group-based influences, such as ingroup loyalty or outgroup bias, factors known to shape defending and aggressive behaviors in peer contexts (Carmona-Rojas et al., 2023). For example, a student may consistently defend a particular peer not due to stable prosocial traits, but because of friendship or a shared group identity, or may follow a peer based on a close personal bond, irrespective of broader tendencies like moral disengagement or susceptibility to peer norms. Fourth, the unequal distribution of participants across trajectory groups, particularly in the *Increase* and *Decrease in Pro-bullying* profiles, may have limited the statistical power to detect nuanced effects and constrained subgroup analyses, such as gender comparisons. Lastly, the absence of data from earlier developmental stages precludes conclusions about whether these trajectories reflect long-standing behavioral tendencies or more recent changes. The study was also conducted in a specific cultural and educational context, which may affect the generalizability of the findings. In addition, all schools adhered to the regional convivencia framework. However, the fidelity and intensity of its implementation, which may have influenced school climate and, in turn, patterns of bystander behavior, were not assessed. These findings carry important implications for intervention design. Preventive efforts should go beyond addressing individual attitudes and include strategies aimed at strengthening moral reasoning, promoting autonomous decision-making, and challenging conformity to peer group norms that validate aggression. Moreover, supporting adolescents in developing the social-emotional skills necessary to resist peer pressure, especially during transitional school periods, may be critical for preventing escalation into stable pro-bullying patterns.

## Conclusion

Research on bystander behavior in bullying has often overlooked how roles develop over time and what mechanisms explain shifts between them. Addressing this gap, the present study identified three trajectories across adolescence, *Sustained Defending*,* Increase Pro-bullying*, and *Decrease Pro-bullying*, and showed that stability and change in bystander behavior are shaped by both timing and socio-moral processes. Most adolescents maintained consistent defending, but a late decline in defending emerged between 9^th^ and 10^th^ Grade, while smaller groups either escalated into pro-bullying behaviors or moved away from it. Passivity proved decisive: in the *Increase Pro-bullying* profile it preceded the shift from avoidance to active reinforcement of aggression, whereas in the *Decrease Pro-bullying* profile it co-occurred with reductions in pro-bullying and gains in defending, underscoring its ambivalent value. Persistently high moral disengagement combined with rising peer conformity coevolved with pro-bullying, while parallel declines in both factors aligned with a shift toward defending. These findings provide empirical evidence that socio-moral processes and behavioral trajectories co-evolve, supporting the reconceptualization of bystander behavior as a developmental process shaped by intrapersonal and interpersonal forces, and advancing understanding of adolescence as a period of dynamic role negotiation in peer contexts.

## Supplementary Information

Below is the link to the electronic supplementary material.

Supplementary Material 1

## Declarations

### Data Sharing and Declaration

The datasets generated and/or analyzed during the current study are not publicly available but are available from the corresponding author on reasonable request.
