# Supplementary material for: Trajectories of Bystander Behaviors in Bullying during Secondary Education: the Role of Moral Disengagement and Conformity To Peer Pressure
Source: J Youth Adolesc. 2025 Oct 22;55(1):168–83. doi: 10.1007/s10964-025-02276-8 (PMC12816034; doi:10.1007/s10964-025-02276-8)
Supplement: Supplementary file 1 — Supplementary Material 1 [file 10964_2025_2276_MOESM1_ESM.docx]

**Trajectories of Bystander Behaviors in Bullying During Secondary Education: The Role of Moral Disengagement and Conformity to Peer Pressure**

**Online Resource**

**Table S1**

*Item-level Missing Data by Variable and Wave*

| Variables | % Missing |
| --- | --- |
| 1. Pro-bully bystander behavior (T1) | 2.1 – 3 |
| 1. Pro-bully bystander behavior (T2) | 1.9 – 2.3 |
| 1. Pro-bully bystander behavior (T3) | 0.7 – 7.8 |
| 1. Passive bystander behavior (T1) | 1.8 – 3.8 |
| 1. Passive bystander behavior (T2) | 0.9 – 2 |
| 1. Passive bystander behavior (T3) | 2.4 – 3 |
| 1. Defender bystander behavior (T1) | 1.9 – 2.9 |
| 1. Defender bystander behavior (T2) | 1.2 – 1.7 |
| 1. Defender bystander behavior (T3) | 2 – 2.8 |
| 1. Moral disengagement (T1) | 0.2 – 1.1 |
| 1. Moral disengagement (T2) | 0.2 – 12 |
| 1. Moral disengagement (T3) | 0.2 – 0.9 |
| 1. Conformity to peer pressure (T1) | 0.3 – 1.2 |
| 1. Conformity to peer pressure (T2) | 0.2 – 0.7 |
| 1. Conformity to peer pressure (T3) | 0.1 – 0.4 |

**Table S2**

*Group Comparisons of Study Variables by Number of Data Collection Waves Attended*

| Variables | Attend 2 times | | | Attend 3 times | | | *t*-Student | |
| --- | --- | --- | --- | --- | --- | --- | --- | --- |
|  | *n* | *M* | *SD* | *n* | *M* | *ST* | *t* | *d* |
| 1. Pro-bully bystander behavior (T1) | 394 | 1.29 | 0.59 | 335 | 1.33 | 0.72 | -0.91 | - |
| 1. Pro-bully bystander behavior (T2) | 226 | 1.42 | 0.90 | 333 | 1.40 | 0.83 | 0.27 | - |
| 1. Pro-bully bystander behavior (T3) | 506 | 1.27 | 0.68 | 359 | 1.33 | 0.71 | -1.16 | - |
| 1. Passive bystander behavior (T1) | 381 | 2.65 | 1.21 | 326 | 2.87 | 1.33 | -2.32* | 0.17 |
| 1. Passive bystander behavior (T2) | 226 | 2.86 | 1.40 | 339 | 2.93 | 1.42 | -0.57 | - |
| 1. Passive bystander behavior (T3) | 496 | 3.03 | 1.32 | 348 | 3.23 | 1.40 | -2.07 | - |
| 1. Defender bystander behavior (T1) | 398 | 5.09 | 1.39 | 336 | 5.13 | 1.41 | -0.37 | - |
| 1. Defender bystander behavior (T2) | 233 | 4.93 | 1.49 | 328 | 4.79 | 1.58 | 1.08 | - |
| 1. Defender bystander behavior (T3) | 500 | 4.42 | 1.56 | 349 | 4.47 | 1.55 | -0.42 | - |
| 1. Moral disengagement (T1) | 416 | 1.74 | 0.55 | 274 | 1.79 | 0.57 | -1.33 | - |
| 1. Moral disengagement (T2) | 418 | 1.54 | 0.59 | 248 | 1.60 | 0.63 | -1.13 | - |
| 1. Moral disengagement (T3) | 494 | 1.53 | 0.53 | 310 | 1.63 | 0.59 | -2.39 | - |
| 1. Conformity to peer pressure (T1) | 492 | 1.47 | 0.51 | 300 | 1.55 | 0.54 | -2.27 | - |
| 1. Conformity to peer pressure (T2) | 527 | 1.49 | 0.62 | 327 | 1.47 | 0.62 | 0.36 | - |
| 1. Conformity to peer pressure (T3) | 520 | 1.44 | 0.55 | 356 | 1.51 | 0.65 | -1.65 | - |

*Note.* **p* < .05.
